# Peripheral immune markers and antipsychotic non-response in psychosis

**DOI:** 10.1016/j.schres.2020.12.020

**Published:** 2021-04

**Authors:** Daniela Enache, Naghmeh Nikkheslat, Dina Fathalla, B. Paul Morgan, Shôn Lewis, Richard Drake, Bill Deakin, James Walters, Stephen M. Lawrie, Alice Egerton, James H. MacCabe, Valeria Mondelli

**Affiliations:** a King's College London, Institute of Psychiatry, Psychology and Neuroscience, Department of Psychological Medicine, London, UK; b Department of Neurobiology, Care Sciences and Society, Division of Neurogeriatrics, Karolinska Institutet, Stockholm, Sweden; c Dementia Research Institute, Division of Infection and Immunity, School of Medicine, Cardiff University, Cardiff, UK; d Department of Psychiatry, University of Manchester, Manchester, UK; e MRC Centre for Neuropsychiatric Genetics, Cardiff University, Cardiff, UK; f Department of Psychiatry, University of Edinburgh, Edinburgh, UK; g King's College London, Institute of Psychiatry, Psychology and Neuroscience, Department of Psychosis Studies, London, UK; h National Institute for Health Research (NIHR) Mental Health Biomedical Research Centre, South London and Maudsley NHS Foundation Trust, King's College London, UK

**Keywords:** Antipsychotic response, Schizophrenia, Cytokines, Inflammation, Negative symptoms, Complement system

## Abstract

**Background:**

Peripheral immune markers have previously been linked to a poor response to antipsychotic medication and more severe negative symptoms at the onset of psychosis. The present study investigated the association of blood cytokines and complement markers with the presence of antipsychotic non-response and symptom severity in patients with psychosis.

**Methods:**

This cross-sectional study recruited 94 patients with schizophrenia and other psychoses, of whom 47 were defined as antipsychotic responders and 47 as antipsychotic non-responders. In all subjects we measured plasma levels of cytokines (IL-1β, IL-2, IL-4, IL-6, IL-8, IL-10, IL-12p70, IL-13, TNF-α, and IFN-γ), complement markers (C1-inhibitor, C3, C4, C3a, C3b, Bb, factor D, C5a, terminal complement complex) and high sensitivity C-reactive protein (hsCRP). Symptom severity was recorded using the Positive and Negative Syndrome scale for Schizophrenia (PANSS). Binary logistic regression tested each immune marker as predictor of response status while covarying for relevant socio-demographic variables. Correlation analyses tested the association between immune markers and the severity of symptoms.

**Results:**

Interleukin (IL)-8 significantly predicted antipsychotic non-response (OR=24.70, 95% CI, 1.35–453.23, p = 0.03). Other immune markers were not associated with antipsychotic response. IL-6, IL-8, IL-10 and TNF-α significantly positively correlated with negative psychotic symptoms.

**Conclusions:**

Higher levels of IL-8 are associated with a poor response to antipsychotic treatment. Increased cytokines levels are specifically associated with more severe negative symptoms in patients with schizophrenia and other psychoses.

## 1 Introduction

Approximately one third of patients with schizophrenia meet criteria for treatment resistance (Meltzer, 1997). The biological mechanisms that underlie non-response to antipsychotics are unclear (Gillespie et al., 2017), but there is growing evidence that an over-activity of the immune system may play a role (Zhang et al., 2004; Mondelli et al., 2015; Miller and Goldsmith, 2017). Epidemiological studies have linked prenatal maternal infections or inflammation during pregnancy with the occurrence of schizophrenia in offspring (Miller and Goldsmith, 2017; Severance et al., 2014). Systemic autoimmune diseases, including lupus erythematosus, autoimmune thyroid disorders and celiac disease, are also associated with increased incidence of psychotic symptoms (Cullen et al., 2018). This may be because peripheral inflammation increases the blood brain barrier permeability leading to increased inflammation in the brain (Khandaker et al., 2015). Moreover, genome wide association studies suggest an association between schizophrenia and genes involved in the immune function such as major histocompatibility complex locus genome (Schizophrenia Psychiatric Genome-Wide Association Study (GWAS) Consortium, 2011) and complement genes (Sekar et al., 2016).

Several systematic reviews and meta-analyses present evidence for altered peripheral cytokines in patients with first episode psychosis or schizophrenia, indicating increased immune activation (Frydecka et al., 2018; Goldsmith et al., 2016; Miller et al., 2011; Pillinger et al., 2018; Potvin et al., 2008; Zajkowska and Mondelli, 2014). One meta-analysis showed that acutely relapsed patients with schizophrenia have increased levels of peripheral interleukins (IL) including IL-6, IL-8, tumor necrosis factor alpha (TNF-α), interferon gamma (IFN-γ) but reduced levels of IL-10 when compared with healthy controls (Miller et al., 2011). Another meta-analysis found that IL-8 was increased in patients with established schizophrenia, but not in patients with first episode psychosis (Frydecka et al., 2018). In post-mortem brain tissue, markers of microglia activation, IFN-γ and TNF-α are also elevated (Mondelli et al., 2017; Trepanier et al., 2016). All these studies suggest the existence of an increased peripheral and central inflammation in patients with established schizophrenia, and an association with treatment response.

The role of the complement system, component of the immune response, in the pathophysiology of treatment resistant schizophrenia is largely unknown (Nimgaonkar et al., 2017). While serum levels of C3 and C4 are increased in drug free patients with established schizophrenia (Maes et al., 1997), serum levels of C3 are decreased in medicated patients with established schizophrenia (Wong et al., 1996), suggesting a possible effect of antipsychotic treatment on these markers. Moreover, abnormal peripherals levels of complement proteins such as C4, C1, C1q, C2, C3, C4 and the terminal complement complex (TCC) have been reported in patients with schizophrenia or first episode psychosis (Kopczynska et al., 2017; Nimgaonkar et al., 2017; Sekar et al., 2016). Research focussing on the association between complement markers and severity of symptoms has reported a positive association between serum levels of C3 and C4 and two items from the Positive and Negative Syndrome scale for Schizophrenia (PANSS) negative symptom scale, referring to active social avoidance and deficient attention, but no associations between complement markers and PANSS positive symptoms (Mayilyan et al., 2008). Our recent paper in a small sample of patients with first episode psychosis showed increased C4 levels in patients with worse clinical outcome at 1-year follow-up when compared with those showing an improvement and overall better treatment response (Mondelli et al., 2020).

Some studies on established schizophrenia have also suggested that abnormal levels of peripheral cytokines may be linked to the severity of negative symptoms and cognitive impairment. Increased levels of TNF-α (Goldsmith et al., 2018b; Xiu et al., 2012), IL-1β (Zhu et al., 2018), IL-3 (Xiu et al., 2015) and IL-18 (Xiu et al., 2012) have been associated with more severe PANSS negative symptoms (Goldsmith et al., 2018b; Zhu et al., 2018), PANSS general symptoms (Xiu et al., 2015, Xiu et al., 2012) or PANSS depression item (Xiu et al., 2012). However, other studies found no correlation between serum IL-6 (Goldsmith et al., 2018b), TNF-α and PANSS score or cognitive performance (Hori et al., 2017). IL-2, known as an anti-inflammatory cytokine, was negatively associated with PANSS positive symptoms and positively associated with PANSS negative (Noto et al., 2015) and PANSS cognitive items (Tan et al., 2015). Our previous research showed that higher serum levels of IL-6 and IFN-γ predicted a poor response to antipsychotic medication after 12 weeks of treatment in patients with first episode psychosis (Mondelli et al., 2015). No study has yet investigated the association between a wide range of peripheral immune markers and antipsychotic response in patients with established psychotic disorders.

An increasing number of clinical trials have been recently conducted using anti-inflammatory drugs in patients with psychosis with the aim of finding alternative treatment strategies for those patients who have not responded to conventional antipsychotic treatment. The findings from these trials have been so far inconsistent (Sommer et al., 2014) and this has partially been ascribed to lack of proper stratification and lack of biomarkers able to identify those who would be more likely to benefit from such treatments. Therefore, the identification of immune biomarkers of response to antipsychotic treatment is particularly important to guide and improve the efficacy of future clinical trials testing anti-inflammatory treatments in patients suffering with psychotic disorders.

The specific aims of this study were to investigate 1) whether levels of immune markers are associated with poor treatment response in patients with established psychotic disorders, and 2) whether raised levels of immune markers are associated with increased symptom severity. We hypothesised that 1) increased levels of pro-inflammatory markers would be associated with status of antipsychotic non-response, and 2) increased pro-inflammatory markers would be associated with more severe negative symptoms.

## 2 Methods

### 2.1 Subjects

A total of 94 patients with schizophrenia and related psychoses (47 antipsychotic-responders and 47 antipsychotic non-responders) were included as part of a cross-sectional study performed by the Schizophrenia: Treatment Resistance and Therapeutic Advances (STRATA) consortium. Patients were recruited across 4 university research sites in the UK: King's College London, University of Manchester, Cardiff University and University of Edinburgh. Inclusion criteria were: (a) age 18–65, (b) DSM-5 schizophrenia or schizophreniform disorder and (c) able to understand the study-related procedures and provide capacitous, informed consent. Exclusion criteria were: (a) pregnancy, (b) any episode of severe head injury involving loss of consciousness for more than 5 min, (c) meeting the ICD criteria for substance misuse or psychotic disorder due to substance misuse, (d) treatment with clozapine in the last 3 months prior inclusion in the study. The study was approved by the South East Coastal Research Ethics Committee, United Kingdom. All participants provided written consent prior to performing any study-related activity.

### 2.2 Clinical assessment

Participants underwent an initial interview to collect demographic data, structured assessment of medical history and Mini International Neuropsychiatric Interview to confirm the diagnosis of schizophrenia and related psychoses. Symptom severity was assessed on the PANSS (Kay et al., 1987), and the Clinical Global Impression Scale (CGI-SCH) (Haro et al., 2003). Adherence to antipsychotic medication was assessed using the Kemp Compliance Rating Scale (Kemp et al., 1998).

Treatment responders were defined as having (i) treatment with only one antipsychotic drug since onset, or that if there were any treatment changes then that these were due to adverse effects and not for non-response; (ii) CGI-SCH score of <4; (iii) PANSS total score of <60 (Howes et al., 2017) (iv) compliance rating scale score of >3 (Kemp et al., 1998). The treatment non-responders group was defined as having (i) documented treatment with at least two antipsychotics for more than 4 weeks each antipsychotic above the minimum therapeutic dose as defined by the British National Formulary; (ii) CGI-SCH score > 3; (iii) PANSS total score of at least 70; (iv) compliance rating scale score > 3 (Howes et al., 2017).

### 2.3 Plasma immune markers

Plasma cytokines, complement markers and high sensitivity C-reactive protein (hsCRP) were measured blind to treatment response status.

Blood samples were collected and centrifuged within 1 h of collection at 1300–2000*g* for 10 min. Aliquots of 500ul plasma were stored at −80 °C until time of the analysis. Candidate cytokines were measured in duplicate using Meso Scale Discovery (MSD) V-plex immunoassays (MSD, Maryland, USA) according to the manufacturer's instructions. The standard Pro-inflammatory Panel 1 (human) kit was used to measure IFN-γ, IL-1β, IL-2, IL-4, IL-6, IL-8, IL-10, IL-12p70, IL-13, and TNF-α. The inter-assay coefficient of variations was <10%.

Complement markers were chosen from classical (C1inhibitor (C1inh), C3, C4, C3a, inhibitor C3b (iC3b)), alternative (b fragment of factor B (Bb), factor D (FD), C3a, C3b) and lytic (C5a, terminal complement complex (TCC)) pathways. Levels of C5a, TCC, Bb, C3a and C3b were measured using customised MSD V-plex assays. Plates were pre-coated using the following commercial antibodies: Mab2952 (C5a) – Hycult, HM2079, aE11 (TCC) – Hycult, HM2167-1A, NeoBb (Bb) – Quidel, A252, 2991 (C3a) – Hycult, HM2074, BH6 (iC3b) – Hycult, HM2168. Pre-coated plates were then blocked in 150 μl/well of 3% BSA in PBS at room temperature for 2 h on a shaker (600 rpm). The block was discarded and 25 μl/well of standards were added in duplicate then diluted (1 in 5) down the plate to generate standard curves. Standards were prepared in 1% BSA + 10 mM EDTA-PBS and starting concentrations for the standards were as follows: Bb 2μg/ml, C3a 5μg/ml, C5a 2μg/ml, iC3b 2μg/ml, TCC 2μg/ml.

FD and C1inh were measured in duplicate using enzyme-linked immunosorbent assays (ELISA). For FD, plates were coated with anti-FD mAb (Hycult - HM2258B). Anti-C1inh mAb used for coating were produced in-house according to previously published methods (Gasque and Morgan, 1996).

hsCRP, complement C3 and C4 were measured at King's College Hospital Viapath laboratories using turbidimetric methods. For hsCRP, C3 and C4 the lowest concentration that can be distinguished from zero is 0.1 mg/l for the high-sensitivity method. Results below this are reported as <0.1 and considered under the detection threshold.

### 2.4 Statistical analysis

Data were analysed using the Statistical Package for Social Sciences version 24.0 (SPSS Inc., USA). Logarithmic transformation was applied to all plasma cytokines and complement measurements to normalise distribution before running statistical analyses. IL-1β and C3a were excluded from statistical analyses as a large part of the values received from the laboratory fell below the detectable threshold (IL-1β levels were below the detectable threshold in 28 samples and C3a levels below the detectable threshold in 68 samples).

In order to test the association between each inflammatory marker and the response to treatment, we used binary logistic regression analysis using the dichotomous variable “treatment responders/treatment non-responders” as dependent variable and covarying for age, sex, body mass index (BMI) and current smoking status. Each logarithmic transformed inflammatory marker was separately introduced in the model increasing the available observations for each marker. The selection of covariates was based on literature review regarding their possible role on inflammatory markers. In our sample BMI was significantly higher among non-responders (see Table 1). Moreover, we performed Pearson and Spearman correlations as appropriate between chosen covariates and inflammatory markers. BMI, age and smoking were correlated with several inflammatory markers.

**Table 1:** Socio-demographic and clinical characteristics of the sample.

Characteristics	Responders (n = 47)	Non-responders (n = 47)	p-Values
Age	29.6 (9.0)	29.3 (8.2)	0.9
Male n (%)	39 (83)	38 (81)	0.8
Education years	13.1 (2.5)	13.1 (2.8)	0.9
Currently smoker n (%)	28 (60)	30 (64)	0.6
BMI/n	27.2 (4.7)/41	30.2 (5.2)/36	**0.01**
Cannabis use n (%)	34 (72)	39 (83)	0.3
Single n (%)	42 (89)	39 (83)	0.1
Unemployed n (%)	34 (72)	37 (79)	0.5
Local housing authority n (%)	18 (38)	24 (51)	0.3
PANSS total score	52.9 (5.5)	86.9 (9.4)	**<0.001**
PANSS positive score	12.2 (3.2)	22.6 (3.5)	**<0.001**
PANSS negative score	13.5 (3.3)	20.9 (4.7)	**<0.001**
PANSS general symptoms	27.2 (3.5)	43.4 (5.9)	**<0.001**
Duration from first psychotic symptoms (years)	4.5 (7.0)	5.4 (6.2)	0.5
Number hospitalizations median (range)	1 (0–4)	1 (0–4)	0.2
Chlorpromazine equivalent dose	461.8 (248.9)	508.3 (354.4)	0.5

Values are mean (SD) unless otherwise specified.

p-Values for the comparisons between excluded and included patients were based on *t-*test, Mann-Whitney, chi-squared tests as appropriate. Bold indicate significant p values.

n: number of subjects, BMI: body mass index, PANSS: positive and negative syndrome Scale.

We ran partial correlation analyses to test the association between immune markers (IFN-γ, IL-2, IL-4, IL-6, IL-8, IL-10, IL-12p70, IL-13, TNF-α, C1inh, C3, C4, iC3b, Bb, FD, C3b, C5a, TCC) and severity of specific symptoms including PANSS positive score, PANSS negative score, and PANSS total score. The partial correlations were adjusted for BMI; age, gender and smoking. In order to control for multiple comparisons, a Benjamini and Hochberg approach was employed with a false discovery rate (FDR) set at 0.25 (Hochberg and Benjamini, 1990). This approach provides better control of type I error rates when conducting multiple hypothesis tests as compared to more conservative approaches (Benjamini and Hochberg, 1995; Holm, 1979).

There were no differences in recruitment or in sociodemographic characteristics of the patients across sites (see Supplementary Table 1). Furthermore, blood samples and processing of the samples were performed across all the centres using a common protocol and standard operating procedures which ensured consistency across the sites. All the assays were centralised and performed in the same laboratory depending on the specific markers. Cytokines for all sites were all measured on the same MSD machine at King's College London. hs-CRP, C3 and C4 for all sites were measured at King's College Hospital Viapath laboratory. The other complement markers for all sites were measured at Cardiff University site. Therefore, we consider that adjusting for each site is unnecessary for these particular analyses.

## 3 Results

Characteristics of the study population are shown in Table 1. The treatment-responder and treatment non-responder groups did not differ significantly in age, sex or duration of illness. BMI was higher in the non-responders compared with the responder group. As expected, PANSS scores were higher in the treatment non-responder group. In terms of medical comorbidities which may have affected the immune system, our patients had history of: adulthood asthma (n = 9), childhood asthma (n = 2), eczema (n = 1), rhinitis (n = 1), HIV under treatment (n = 1), undiagnosed fever-like glandular syndrome (n = 1), diabetes (n = 3), psoriasis (n = 1), past meningitis (n = 1). This is typical of patients with psychosis who tend to present higher medical comorbidity compared with general population.

### 3.1 Immune markers as predictors of treatment non-responder status

Binary logistic regression of individual inflammatory markers adjusted for age, gender, BMI and smoking status found IL-8 significantly predicted antipsychotic non-response (Odds ratios (OR) 24.704, 95% confidence interval (95% CI): 1.35–453.23) with higher levels of IL-8 in non-responders compared with responders. The remaining inflammatory markers were not significantly associated with antipsychotic response in this analysis (see Table 2; un-adjusted odds ratios are reported in Supplementary Table 2).

### 3.2 Relationships between inflammatory markers and symptom severity

Correlation analyses subsequently tested the associations between symptom severity (PANSS total score, PANSS positive score, PANSS negative score) and immune markers.

**Table 2:** Binary logistic regression predicting response to treatment (Responders vs Non-Responders) covarying for age, gender, BMI and current smoking status.

Predictor	B value	S.E.	p value	Exp(B)	Benjamini-Hochber critical values	95%CI
**IL-8**	**3.207**	**1.48**	**0.031**	**24.704**	**0.014**	**1.346–453.226**
IL-10	1.654	1.05	0.114	5.227	0.03	0.671–40.731
IL-13	1.089	0.70	0.118	2.972	0.04	0.758–11.648
IFN-γ	−1.384	0.94	0.139	0.250	0.06	0.040–1.570
IL-6	1.286	0.92	0.161	3.620	0.07	0.600–21.840
TCC	0.501	0.79	0.526	1.651	0.08	0.351–7.769
C1inhib	0.839	1.34	0.530	2.314	0.10	0.168–31.788
C5a	0.349	0.59	0.552	1.418	0.11	0.449–4.479
FD	1.233	2.08	0.554	3.431	0.12	0.058–202.766
IL12p70	−0.412	0.75	0.585	0.663	0.14	0.151–2.902
Bb	−0.771	1.48	0.603	0.463	0.15	0.025–8.447
IL-4	−0.281	0.66	0.671	0.755	0.17	0.207–2.757
iC3b	0.438	1.06	0.680	1.550	0.18	0.193–12.465
TNF-α	0.857	2.27	0.706	2.356	0.19	0.027–202.382
IL-2	0.179	0.66	0.786	1.196	0.21	0.328–4.365
C3	−0.770	3.31	0.816	0.463	0.22	0.001–305.627
hsCRP	−0.034	0.77	0.967	0.967	0.24	0.831–1.125
C4	−0.036	1.66	0.983	0.964	0.25	0.038–24.770

IL: interleukin, IFN: interferon, TCC: terminal complement complex, C1inhib: inhibitor of the complement C1 complex to prevent spontaneous activation, C5a: complement component 5a, FD: factor D, Bb: activated factor B, TNF: tumor necrosis factor, hsCRP: high-sensitive C reactive protein. B – value: coefficient for the constant (or “intercept”), SE: standard error around B-coefficient. Bold indicate statistically significant findings.

Exp (B): exponentiation of the B coefficient, represent Odds ratio, 95% CI: 95% confidence interval.

**Fig. 1:**
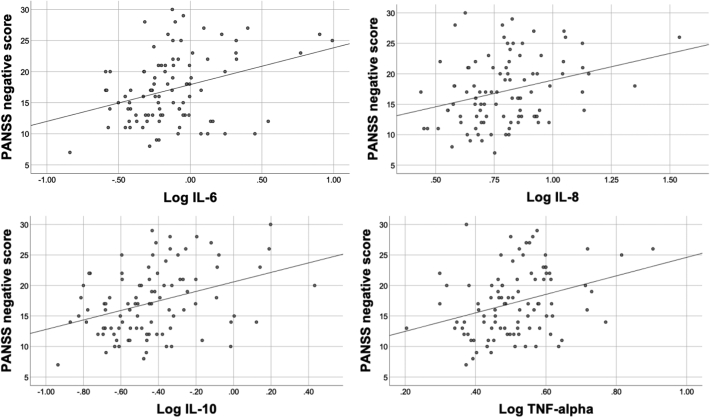
Associations between IL-6, IL-8, IL-10 and TNF-α and negative symptoms in the whole cohort.

IL-8, IL-10 and TNF-α positively correlated with PANSS negative symptom score (IL-8, r = 0.27, p = 0.02; IL-10, r = 0.38 p = 0.001; TNF-α, ρ = 0.27, p = 0.002; see Fig. 1). IL-6 positively correlated with PANSS negative score (r = 0.27, p = 0.03), but was not significant after correcting for multiple comparison. There was no significant correlation between these immune markers and PANSS positive score or PANSS total score. No other correlation survived multiple comparisons correction.

In the non-responders group IL-6, IL-10, TNF-α positively correlated with PANSS negative score (IL-6 r = 0.46, p = 0.01; IL-10 r = 0.57, p = 0.001, TNFα r = 0.41, p = 0.02). C4 positively correlated with PANSS negative score (r = 0.38, p = 0.03), but it was not significant after correcting for multiple comparison.

TCC negatively correlated with PANSS positive score (r = −0.48, p = 0.01). C5a negatively correlated with PANSS general symptoms score (C5a r = −0.45, p = 0.01).

In the responders group IL-8 positively correlated with PANSS negative score (IL-8 r = 0.34, p = 0.04). A negative correlation was found between Bb and TCC and PANSS negative score (Bb r = −0.35, p = 0.04, TCC ρ = −0.38, p = 0.03). IL-12p70 negatively correlated with PANSS positive symptom (r = −0.41, p = 0.01). There was no significant correlation between immune markers and general symptom. No other correlation survived multiple comparisons correction.

## 4 Discussion

This study shows that higher levels of IL-8 were associated with poor antipsychotic response in patients with established psychotic disorders. Across the whole sample, IL-8, TNF-α and IL-10 positively correlated with negative symptom severity, but not with positive or general symptom scores. In the non-responders, IL-6, TNF-α and IL −10 strongly and positively correlated with negative symptom severity, while TCC and C5a negatively correlated with the positive symptoms and general symptom severity, respectively. In the treatment-responder group IL-8 positively correlated with negative symptoms severity, while factor B (Bb) and TCC correlates negatively with negative symptoms severity. Also, in the responders group IL-12p70 correlated negatively with positive symptoms severity.

Our results are in line with previous findings in first-episode psychosis of an association between increased levels of pro-inflammatory cytokines and poor treatment response (Mondelli et al., 2015). However, the specific immune markers associated with treatment resistance appeared different from those observed at onset of psychosis. In first-episode psychosis a poor response to treatment was associated with increased levels of IL-6 and IFN-γ (Mondelli et al., 2015) whereas in the current sample of patients with established schizophrenia the association was related to increased levels of IL-8. The difference in immune markers could be partially due to the different stages of illness that have been investigated in the two studies. It is interesting that the immune signal linked to poor antipsychotic response in our sample appears more specifically represented by levels of IL-8. This appears in agreement with a previous study (Zhang et al., 2004), who showed that high baseline levels of IL-8 in patients with established schizophrenia were associated with less improvement after 12 weeks of treatment with either risperidone or haloperidol. Furthermore, a recent meta-analysis showed higher cerebrospinal fluid (CSF) levels of both IL-8 and IL-6 in patients with schizophrenia spectrum disorder when compared with healthy controls, supporting the idea that the elevation of the cytokines observed in peripheral blood may correspond to also a central pro-inflammatory effect (Gallego et al., 2018). However, other studies found no significant association between serum levels of IL-8 and response to treatment in patients with schizophrenia (Erbağci et al., 2001; Maes et al., 2002; Reale et al., 2011).

The association between pro-inflammatory cytokines and the severity of negative symptoms in our sample is broadly consistent with previous studies. Similar associations have been reported in patients with established schizophrenia for TNF-α and IL-6 (Goldsmith et al., 2018b), in patients with first episode psychosis for IFN-γ (Mondelli et al., 2015) and more recently in individuals at ultra-high risk of psychosis with TNF-α and IL-6 (Goldsmith et al., 2018a). The link between immune activation/inflammation and negative symptoms has been hypothesised to be related to an effect of prolonged immune activation on the brain leading to a progressive brain volume loss, reduced neurogenesis, neuroplasticity and synaptic pruning (Guo et al., 2015; Nimgaonkar et al., 2017). Supporting this hypothesis, we have previously shown high levels of IL-6 to be associated with smaller hippocampal volume in patients with first episode psychosis (Mondelli et al., 2011). A more recent study also suggested that brain volume loss in schizophrenia is associated with a genetic predisposition to produce more IL-1β (Muller, 2018).

Although IL-6 and TNF-α have been already previously suggested as a possible important target for patients with treatment-resistant psychosis and with prevalent negative syndrome (Goldsmith et al., 2018b; Mondelli et al., 2015), the findings of increased IL-10 were more unexpected. Interleukin-10 is an important anti-inflammatory cytokine, which has been previously described to revert depressive-like behaviour in IL-10 knock-out mice (Mesquita et al., 2008). The increased levels of IL-10 could be interpreted as possible compensating mechanism of the organism to reduce these symptoms. Alternatively, increased IL-10 levels could represent an epiphenomenon, where, for example, IL-10 could be increased following exposure to infections, which are more likely to happen in patients with negative symptoms who present lower self-care and unhealthier lifestyle (Miller and Goldsmith, 2019).

Another interesting finding is that the association between immune markers and negative symptoms seems to differ between responders and non-responders. We found that in non-responder patients, pro-inflammatory (IL-6, TNF-α) and anti-inflammatory (IL-10) cytokines were associated with increased severity of negative symptoms, while terminal complement complex (TCC) negatively correlated with positive symptoms severity and C5a negatively correlated with general symptom severity. C5a is a protein belonging to the lytic pathway, it is formed after the cleavage of C5 into C5a and C5b and it has a chemotactic role and activates mast cells (Mayilyan et al., 2008; Woo et al., 2020). The terminal complement components (TCC) is a key protein belonging to the lytic pathway of the complement, and it forms membrane attack complexes (MAC) which disrupt cell membranes and cause cell death (Mayilyan et al., 2008). The reduced levels of both C5a and TCC suggest that the activity of the lytic pathway of complement may be reduced in non-responder patients with more severe positive and general symptoms (Woo et al., 2020).

In treatment-responder patients we found that higher levels of IL-8 and lower levels of factor B and TCC were associated with more severe negative symptoms. In addition, IL-12p70 negatively correlated with PANSS positive symptom. IL-8 (or C-X-C motif chemokine ligand 8-CXCL8) is a chemokine secreted by a variety of cells with immunological roles such as monocytes, neutrophils and endothelial cells (Qazi et al., 2011) and has a neutrophil chemoattractant activity and plays a key role in immune cell activation (Yoshimura et al., 1987) in acute (Harada et al., 1994) and chronic inflammation (Qazi et al., 2011), but it also has a role in angiogenesis (Qazi et al., 2011). The increased level of IL-8 suggests an increase activation of monocytes-macrophages in patients with more severe negative symptoms, implying an increase production of monocytes, secretion of chemokines and angiogenesis- maintaining an increased peripheral level of inflammation. In a recent meta-analysis other serum chemokines, such as macrophage inflammatory protein 1beta (MIP1β) and eotaxin-1, have been reported elevated in patients with established schizophrenia compared with normal controls (Frydecka et al., 2018). Factor B is a protein which plays a key role in initiating the alternative pathway of complement (Mayilyan et al., 2008; Slade et al., 2013). The reduced levels of factor B and TCC suggest that the activity of the alternative and lytic pathways of complement may be reduced in treatment-responder patients with more severe negative symptoms, implying a limited propagation of inflammation in these patients.

IL12p70 is secreted mainly by macrophages and plays a key role in activating type 1 T helper (Th1) cells and inducing the production of IFN-γ in the natural killer cells (Kim et al., 2002). The reduced levels of IL12p70 suggest a reduced activation of Th1cells and production of IFN-γ in responder patients with more severe positive symptoms.

The present study has some methodological limitations. First, no assumption regarding causality can be made as the study has a cross sectional design. Second, the effect of antipsychotic treatment on immune markers cannot be ruled out as all patients were on stable antipsychotic medications; however, there were no significant difference in exposure to antipsychotic treatment between treatment responder and non-responder patients. Third, some of the immune markers were under detectable threshold and could not therefore be in included in the analysis. Fourth, the blood samples were collected at different time points during the day and we cannot exclude the effect of diurnal variation on the inflammatory markers. Fifth, we did not excluded patients with acute and chronic inflammatory disease; somatic comorbidities- such as diabetes, asthma, chronic obstructive pulmonary disease, HIV- are common among patients with established schizophrenia (Bitter et al., 2017). We included these patients for a better generalizability of the findings and to increase the power of the analyses. Lastly, our logistic regression analysis shows a strong association between high IL-8 and poor response to antipsychotic treatment (beta value and exponential of B-value –or Odd Ratio) but with a large SE around B value and implicitly a large 95% CI of the OR. The reason for the large SE and 95% CI can be a small sample size, imprecise measure, or just a lot of unexplained variance. Our study has also several strengths. To achieve the sample size, we used a standardised protocol to recruit patients with treatment-responsive and treatment non-responsive psychosis across several centres in the UK. In addition, we assayed a broad range of peripheral inflammatory markers to provide a comprehensive evaluation.

## 5 Conclusions

Increased levels of IL-8 are associated with poor response to antipsychotic treatment in patients with schizophrenia and related psychoses. Increased levels of cytokines are specifically associated with more severe negative symptoms. Future studies should use a longitudinal design to investigate the causal role of immune pathways in development of negative symptoms in patients with established schizophrenia and test the benefit of using immune biomarkers such as IL-8, IL-6, IL-10 and TNF-α to guide anti-inflammatory treatment for severe and refractory negative symptoms.

The following are the supplementary data related to this article.

Supplementary Table 1 Sociodemographic characteristics of the patients across sites. Supplementary Table 1

Supplementary Table 2 Binary logistic regression. Supplementary Table 2

## Data availability

At the time of submission, the data governance frameworks are being put in place to make a fully anonymised version of the data available to the wider research community via TranSMART data sharing platform: https://transmartfoundation.org/, which will be hosted at the MRC eMedLab: https://www.emedlab.ac.uk/. To apply for access to the data, please contact James MacCabe james.maccabe@kcl.ac.uk.

## Role of funding sources

The study was funded by a Stratified Medicine grant to Drs MacCabe, Lewis, Drake, Deakin, Walters, Lawrie and Egerton from the 10.13039/501100000265Medical Research Council (MRC), MR/L011794/1. The views expressed are those of the authors and not necessarily those of the MRC.

## CRediT authorship contribution statement

DE, JM and VM contributed to study design, analysis and interpretation of the data, and writing of the manuscript. NN, DF and BPM contributed to analyses and interpretation of the data, and writing of the manuscript. SL, RD, BD, JW, SML and AE contributed to study design, interpretation of the data, and writing of the manuscript.

## Declaration of competing interest

VM has received research funding from Johnson & Johnson as part of a research program on depression and inflammation. BD has share options in P1vital and has received consultancy fees from Autifony.com. BPM has provided advice on complement to Roche and is a consultant to GlaxoSmithKline; all fees were paid to Cardiff University. JW reported receiving a grant from Takeda Pharmaceuticals outside of the submitted work. In the past three years, SML has received funding for research from Janssen, and personal fees for participating in educational meetings from Janssen and Sunovion. The remaining authors report no conflicts of interest.
