# Supplementary material for: Peripheral immune markers and antipsychotic non-response in psychosis
Source: Schizophr Res. 2021 Apr;230:1–8. doi: 10.1016/j.schres.2020.12.020 (PMC8224180; doi:10.1016/j.schres.2020.12.020)
Supplement: Supplementary Table 1 — Sociodemographic characteristics of the patients across sites. [file mmc1.docx]

**Supplementary Table 1**: Sociodemographic characteristics of the patients across sites

| Site | Number total n=94 (%) | Patient’s age in each site  mean (SD) | Male gender (%) in each site |
| --- | --- | --- | --- |
| King’s College | 35 (37%) | 30.7 (9.5) | 28 (80.0%) |
| Cardiff | 15 (16%) | 27.6 (10.9) | 11 (73.3%) |
| Edinburgh | 16 (17%) | 30.5 (7.7) | 14 (87.5%) |
| Manchester | 28 (30%) | 28.3 (6.05) | 24 (85.7%) |

There was no significant age difference (ANOVA F=0.76, p=0.53) or gender difference across sites (Kruskal Wallis H=1.43 p=0.7)
