# Supplementary material for: Peripheral immune markers and antipsychotic non-response in psychosis
Source: Schizophr Res. 2021 Apr;230:1–8. doi: 10.1016/j.schres.2020.12.020 (PMC8224180; doi:10.1016/j.schres.2020.12.020)
Supplement: Supplementary Table 2 — Binary logistic regression. [file mmc2.docx]

**Supplementary Table 2**: Binary logistic regression

| Predictor | **Model 1** (crude analysis) |  | **Model 1** (adjusting for age, gender, smoking) | p |
| --- | --- | --- | --- | --- |
|  | OR 95%CI | p | OR 95% CI |  |
| IL-2 | 0.95 (0.34-2.67) | 0.95 | 0.95 (0.33-2.72) | 0.9 |
| IL-4 | 0.69 (0.21-2.24) | 0.54 | 0.69 (0.21-2.23) | 0.5 |
| IL-6 | 3.16 (0.77–12.92) | 0.11 | 3.55 (0.82-15.45) | *0.09* |
| IL-8 | 8.09 (0.78-84.33) | 0.08 | 9.71 (0.90-109.55) | *0.07* |
| IL-10 | 3.50 (0.68-18.38) | 0.14 | 3.72 (0.70-19.77) | 0.1 |
| IL12p70 | 0.46 (0.13-1.63) | 0.23 | 0.48 (0.13-1.77) | 0.3 |
| IL-13 | 2.15 (0.72-6.44) | 0.17 | 2.60 (0.79-8.60) | 0.1 |
| TNF-α | 6.08 (0.16-223.77) | 0.32 | 9.46 (0.21-41) | 0.3 |
| IFN-γ | 0.36 (0.08-1.53) | 0.16 | 0.36 (0.08-1.55) | 0.1 |
| Bb | 0.58 (0.05-7.40) | 0.67 | 0.61 (0.05-8.06) | 0.7 |
| C5a | 1.24 (0.47-3.25) | 0.66 | 1.35 (0.48-3.81) | 0.5 |
| iC3b | 1.37 (0.25-7.50) | 0.72 | 1.35 (0.22-8.25) | 0.7 |
| TCC | 1.58 (0.45-5.59) | 0.48 | 1.70 (0.44-6.61) | 0.4 |
| C1inhib | 1.26 (0.16-10.80) | 0.83 | 1.30 (0.14-11.50) | 0.8 |
| FD | 17.00 (0.58-501.90) | 0.10 | 38.90 (0.96-1576.9) | *0.05* |
| C3 | 7.36 (0.05-1122.83) | 0.43 | 6.76 (0.04-1097.24) | 0.4 |
| C4 | 1.20 (0.07-19.24) | 0.90 | 1.13 (0.7-18.94) | 0.9 |
| hsCRP | 1.03 (0.96-1.10) | 0.43 | 1.03 (0.96-1.11) | 0.4 |

IL: interleukin, C3a: complement component 3a, C5a: complement component 5a, FD: factor D, TCC: terminal complement complex, C1inhib: inhibitor of the complement C1 complex to prevent spontaneous activation, Bb: activated factor B, hsCRP: high-sensitive C reactive protein, OR: Odds ratio, 95% CI : 95% confidence interval.
